# A SEM-EDX Study on the Structure of Phenyl Phosphinic Hybrids Containing Boron and Zirconium

**DOI:** 10.3390/gels9090706

**Published:** 2023-09-01

**Authors:** Petru Merghes, Narcis Varan, Gheorghe Ilia, Iosif Hulka, Vasile Simulescu

**Affiliations:** 1 University of Life Sciences ‘‘King Michael I’’ from Timisoara, 119 Calea Aradului, 300645 Timisoara, Romania; petrumerghes@usvt.ro (P.M.); narcisvaran@usvt.ro (N.V.); 2 Faculty of Chemistry, Biology, Geography, West University of Timisoara, 16 Pestalozzi Street, 300115 Timisoara, Romania; gheorghe.ilia@e-uvt.ro; 3 Research Institute for Renewable Energies, Politehnica University Timisoara, 38 Gavriil Musicescu, 300501 Timisoara, Romania; iosif.hulka@upt.ro

**Keywords:** sol–gel synthesis, organic–inorganic hybrids, phenyl phosphinic acid, zirconium, boron, SEM-EDX

## Abstract

The SEM-EDX method was used to investigate the structure and morphology of organic–inorganic hybrids containing zirconium, boron and phosphorus compounds, synthesized by the sol–gel method. We started by using, for the first time together, zirconyl chloride hexa-hydrate (ZrOCl_2_·6H_2_O), phenyl phosphinic acid and triethyl borate as precursors and reagents, at different molar ratios. The obtained hybrids showed a very high thermal stability and are not soluble in water or in organic solvents. As a consequence, such hybrid solid materials are suitable for applications at high temperatures. The obtained hybrids have complex 3D structures and form organic–inorganic networks containing Zr-O-Zr, Zr-O-P and Zr-O-B bridges. Such organic–inorganic networks are also expected to form supramolecular structures and to have many potential applications in different fields of great interest such as catalysis, medicine, agriculture, energy storage, fuel cells, sensors, electrochemical devices and supramolecular chemistry.

## 1. Introduction

The sol–gel process is part of the green chemistry concept, due to the fact that the syntheses take place at room temperature, in mild conditions and by using green solvents (in general, water is used, as in the present work, but sometimes, alcohol or mixtures of alcohol:water at different volume ratios could also be involved). The sol–gel technique is a modern synthesis, which was developed and used in the last decades for obtaining hybrid compounds containing different functional groups (alcoholic, carboxylic, sulphonic groups and so on) [1,2,3]. The sol–gel process is called „Chimie douce” due to the mild conditions in which it takes place and also due to the use of green solvents. Organic–inorganic hybrid materials, sometimes called inorganic polymers or organic–inorganic networks, contain an inorganic part and organic moieties. This makes these hybrid compounds very interesting materials because such networks possess the properties of both, that is, the rigid inorganic backbone and the greater flexibility of organic groups. Due to the surface protons of these organic groups and due to their complex structures, the obtained organic–inorganic hybrids show solid state proton conduction [4,5,6,7]. Furthermore, the organic–inorganic hybrids are not soluble in most organic solvents or in water and have high thermal stability [8]. All of these properties make them very useful for a lot of applications of great interest, as fuel cells, energy storage, different sensors, catalysts, water electrolysis units, other electrochemical devices and so on. By using the sol–gel method, several compounds could be synthesized, starting from phosphonic acids, with a lot of potential applications.

Due to their potential application, such organic–inorganic hybrids containing phosphorus are currently of huge interest. The organic–inorganic hybrid networks are novel materials with promising applications in electrochemical devices and in the fields of catalysis, medicine, or agriculture [1,9,10,11,12,13,14,15,16,17,18,19,20,21,22], as previously mentioned. Hybrids containing phosphorus and zirconium have already been synthesized by using the sol–gel method in different studies, and the results have been published and are available in the literature [1,2,3,4,8,13,14,15,16,17,18,19,20,21]. Alberti et al. [2,4], for example, synthesized different alkyl and phenyl phosphonates containing zirconium, as porous hybrid organic–inorganic materials, by using metal phosphonates as precursors [1,2,3,4]. It was proved that the P/M ratio has a significant influence on the hybrid material porosity (pore shape, pore number, pore size, pore surface distribution and so on) [23,24,25,26,27,28,29,30,31,32].

Other examples of using the sol–gel synthesis are the studies performed and published by Vioux et al. [8,14,16,18,19,31]. They used a two-step sol–gel method for the synthesis of ZrO_2_- and TiO_2_-phenylphosphonate hybrids, as follows:

- The condensation reaction of a phosphonic acid with a metal alkoxide under anhydrous conditions.
- The hydrolysis–condensation reactions of the remaining metal alkoxide groups [27,31,32].

As a consequence, it was observed and proved that M-O-P (metal–oxygen–phosphorus) bridges are obtained during the condensation step and M-O-M (metal–oxygen–metal) bridges are obtained during the hydrolysis–condensation reaction, in the structure of the organic–inorganic hybrids [21,25,26,27,28,29,30,31,32,33,34,35,36,37,38,39,40]. On the other hand, it should be mentioned that the condensation of two or more P-O-H groups, in order to obtain P-O-P bridges, is not possible under the mild conditions used for the sol–gel synthesis [27,29,31,33].

The sol–gel synthesis of hybrid materials containing phosphorus involves the reaction of metal precursors with different phosphonate molecules (phosphonic acids, phosphonic acid derivatives, biphosphonic acids, polyphosphonic acids and derivatives, or phosphonate sodium or ammonium salts in aqueous medium or anhydrous conditions) [27]. These phosphonate molecules are called “coupling molecules” due to their role in the sol–gel process. A templating agent could also be used in order to control and/or change the pore size and, therefore, the materials’ porosity [34,35,36,37,38,39]. Small amounts of some organic additives (for example, different co-polymers containing ethyleneoxide groups such as PEG, with different molar mass and polydispersity, and also non-ionic surfactants or cationic surfactants, cyclodextrins) could be very useful for the improvement of the hybrid’s texture [40].

The structure of the hybrid materials containing phosphorus compounds was studied, as reported in the literature, by several spectroscopic methods, such as FTIR [27,34], X-ray photoelectron spectroscopies [35], SEM, TEM, TG, EDX and XRD.

## 2. Results and Discussion

**Figure 1:**
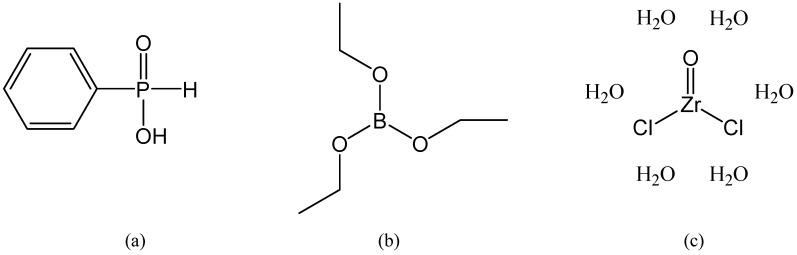
The structure of the used reagents: (**a**) phenyl phosphinic acid, (**b**) triethyl borate B(OEt)_3_; (**c**) zirconyl chloride hexa-hydrate ZrOCl_2_·6H_2_O.

The organic–inorganic hybrid materials synthesized in the present work were obtained by using the sol–gel method, at room temperature, by using water as solvent. It was possible to use a green solvent as water is used because all the precursors and reagents involved in the sol–gel syntheses from the present work are water soluble. Five organic–inorganic hybrids (S1–S5) were synthesized by using zirconyl chloride hexa-hydrate, triethyl borate and phenyl phosphinic acid (Figure 1) for the first time together in a sol–gel process. All the hybrids were synthesized by using the sol–gel method in mild conditions, at room temperature. The products were subsequently analyzed by SEM-EDX technique.

The sol–gel method was chosen to be used in the present work because it is the most efficient, yet also the greenest method (it is part of the green chemistry concept) for obtaining such organic–inorganic hybrids in mild conditions. The efficiency of the sol–gel method as a green synthesis was already proved (including its mechanism) by Vioux et al., Mutin et al., Guerero et al. Clearfield et al., Maillet et al. and Mehring et al. [6,11,12,14,16,18,19,21,31,38,39], as well as many more researchers involved in the fields of organic–inorganic hybrid synthesis and characterization.

All the syntheses (S1–S5) were carried out for 6 h at room temperature, under continuous stirring, by using water as solvent. The used reagents are soluble in water, but the obtained organic–inorganic hybrids are not. This is the first indication that the synthesis takes place, and this is also very helpful for the final separation step (sedimentation, filtration, washing and drying). At the beginning, only the soluble reagents are present, and therefore, in the flask, a homogeneous solution is obtained. When the hybrid appears during the synthesis, due to its insolubility, it leads first to the obtaining of a suspension (with a visible turbidity) and finally a sediment on the bottom of the used flask.

Therefore, the sol–gel syntheses were performed at room temperature, in water, as follows:

- Hybrids S1–S3 were obtained from phenyl phosphinic acid and ZrOCl_2_·6H_2_O, in water.
- Hybrids S4 and S5 were synthesized from phenyl phosphinic acid, ZrOCl_2_·6H_2_O and triethyl borate at different molar ratios, also in aqueous solution.

When the sol–gel synthesis was complete, the obtained products were separated by filtration and subsequently washed several times with water. The used volume of water for washing was minimal. The water, for the sol–gel syntheses and washing, was double distilled just before use. The washing process removed all the possible unreacted (if excessive) precursors, which are highly soluble in water, as mentioned already. The reagents in excess were thereby recovered and the synthesized hybrids were purified. Then, the organic–inorganic hybrids were dried for 6 h at 80 °C in an oven, in order to completely remove the water retained on the products (due to their porosity).

The washing, filtration and drying steps took place consecutively. Considering that the drying took place over 6 h at a temperature of 80 °C, no burning occurred and there was no thermal decomposition. The hybrid compounds were very stable from a thermal point of view. The main aim of the above-mentioned procedures (washing, filtration, drying) was the separation and purification of the insoluble hybrid product, from (if it is the case) unreacted precursors and reagents (usually if in excess). The reagents and precursors are soluble, while the hybrids are not. Finally, the pure dried organic–inorganic hybrid was obtained. This product (for each of the syntheses, S1–S5) was further analyzed by SEM-EDX. On the other hand, the recovered reagents, when necessary (excessive reagents, unreacted precursors and so on), were further separated and purified in order to be used for other syntheses.

The phenyl groups from the structure of phenyl phosphinic acid are very stable in the mild conditions used for the sol–gel process, and they do not react, as we observed in our previous work [8,9,15], except when using a different phosphorus source and therefore a different organic moiety (phenyl phosphonic acid or vinyl phosphonic acid, in these other cases). In other words, the mild conditions used are not sufficient to make the phenyl groups react. Therefore, the organic moiety from the hybrid’s structures was derived from the phenyl phosphinic acid, containing the unreacted and very stable phenyl radicals.

On the other hand, phosphorus, zirconium and boron atoms are always connected via an oxygen atom to the hybrid’s structure and finally lead to the obtaining of a 3D organic–inorganic network. Bridges like P-O-Zr, Zr-O-Zr or B-O-Zr finally lead to the obtaining of the organic–inorganic hybrid, forming the bond between the organic and inorganic moieties. Hybrid S1 was obtained with a phenyl phosphinic acid:ZrOCl_2_ 1:1 molar ratio, while for hybrid S2, phosphinic acid was in excess, and similarly for hybrid S3, zirconium chloride was in excess. The zirconium excess (S3) leads to the formation of more Zr-O-Zr bridges. It is expected that hybrid S2 obtained at a P/Zr 2:1 ratio contains both P-O-Zr and Zr-O-Zr bridges in approximately equal ratio. Hybrid S3 obtained with a zirconium excess at a P/Zr 1:2 ratio has more Zr-O-Zr bridges than hybrids S1 and S2.

In general, it is good to have an excess of phosphorus, and as previously mentioned, the ideal P/M ratio is 2 [11]. When the ratio P/Zr is 2:1, as it is for hybrid S2, it is more likely to obtain both P-O-Zr and Zr-O-Zr bridges in more or less equal ratios. Thus, it is good to have a P/Zr ratio of 2:1 if we want to obtain a hybrid with both P-O-Zr and Zr-O-Zr bridges in an equivalent proportion. But if a hybrid with more P-O-Zr bridges is needed, or one with more Zr-O-Zr bridges is the goal of the sol–gel process, we can change the ratio of the precursors used (for example, an excess of Zr will lead to more Zr-O-Zr bridges). As we can observe, bonds like Zr-O-Zr are also present, but no P-P, Zr-Zr, P-Zr or P-O-P bridges occurred (such bonds could not be obtained in the mild conditions used in the present work for the sol–gel process, as it is also not possible to involve them in the sol–gel reactions or to decompose the phenyl radicals from phenyl phosphinic acid).

Therefore, the excess of one component or the other significantly influenced the structure of the synthesized hybrid and also its properties, first, by changes to the ratio and the number of P-O-Zr and Zr-O-Zr bridges, and then as a consequence, the pore number, the pore shape and, therefore, the entire material porosity. Thus, the organic–inorganic hybrids S1–S3 contain phosphorus and zirconium. In addition, hybrids S4 and S5 synthesized in the presence of triethyl borate, which also contains boron. As already mentioned, phosphorus, zirconium and boron atoms are always connected via an oxygen atom on the hybrid’s structure and finally lead to the obtaining of 3D organic–inorganic networks (because in the mild conditions used for the sol–gel process, no direct bonds like P-P, P-Zr, P-B or Zr-B could be obtained). When boron is added at different ratios (S4 and S5), Zr-O-B bridges are also obtained. As can be observed, only zirconium could be involved in bridges with phosphorus (P-O-Zr), because at room temperature and in the mild conditions used for this sol–gel process, it is not possible to obtain a P-O-B bond. Therefore, while it is expected that there will be competition between boron and zirconium for phosphorus atoms on the hybrids’ structure (for hybrids S4 and S5, both containing boron), in fact, only zirconium could be connected to phosphorus via an oxygen atom (P-O-Zr bridges). Then, no P-O-B and also no B-O-B are obtained. Practically boron is involved only in Zr-O-B bridges. Moreover, phosphorus is contained only in Zr-O-P bridges. In this case of obtaining hybrid materials containing P, Zr and B, an excess of zirconium is needed, as in the case of hybrid S5 synthesized at a phenyl phosphinic acid:ZrOCl_2_:B(OEt)_3_ ratio of 1:2:1. In this way, zirconium could be involved in P-O-Zr, Zr-O-Zr and also in B-O-Zr bridges. If there is an excess of phosphorus and/or boron (or even at equal ratio, as for hybrid material S4), there is expected to be strong competition between phosphorus and boron atoms for zirconium atoms, in order to obtain P-O-Zr or B-O-Zr bridges, or more likely both. The P-O-Zr bridges are in this case the only way to incorporate the organic moieties in the structure of the hybrid materials. When zirconium is excessive (S3 and S5), it is expected to obtain more Zr-O-Zr bridges than P-O-Zr bridges, and as a consequence, these hybrids contain in this case a lower ratio of organic moieties in comparison with the inorganic part. If they are regarded as inorganic co-polymers, there will be more inorganic parts at one organic moiety in their structures. For hybrid S5, the ratio of the organic moieties is even lower, because it also contains boron, which will lead to obtaining B-O-Zr bridges and will be in direct competition with phosphorus and zirconium as well. What is very interesting in all the synthesized hybrids in the present work, is the fact that even the presence of zirconium will lead to obtaining bridges with zirconium. Therefore, the ratios of organic–inorganic moieties are in general higher for the inorganic part.

The phenyl groups from the structure of phenyl phosphinic acid are very stable, as we observed in our previous work when using different phosphonic acids [8,15]. It should be pointed out and given due consideration that the sol–gel syntheses were performed in mild conditions, in water as solvent and at room temperature. In these mild conditions, the phenyl groups do not react, and as a consequence, they will be found on the structure of the synthesized hybrids. Changing the phosphorus source makes a significant difference when compared with our previous work, where we used, for example, phenyl phosphonic acid (it contains two OH groups). The phenyl phosphinic acid used in the present work contains only one OH group directly connected to a phosphorus atom (Figure 1).

**Figure 2:**
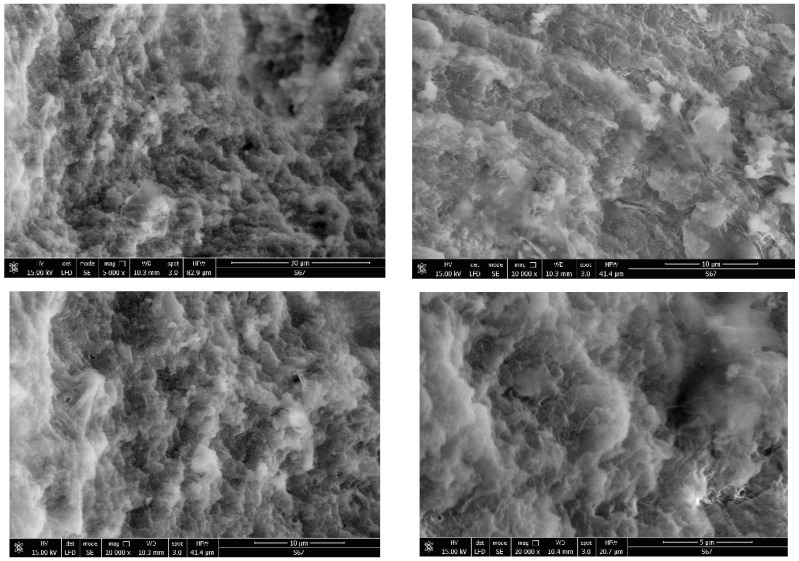
Hybrid material S1 analysed by SEM.

**Figure 3:**
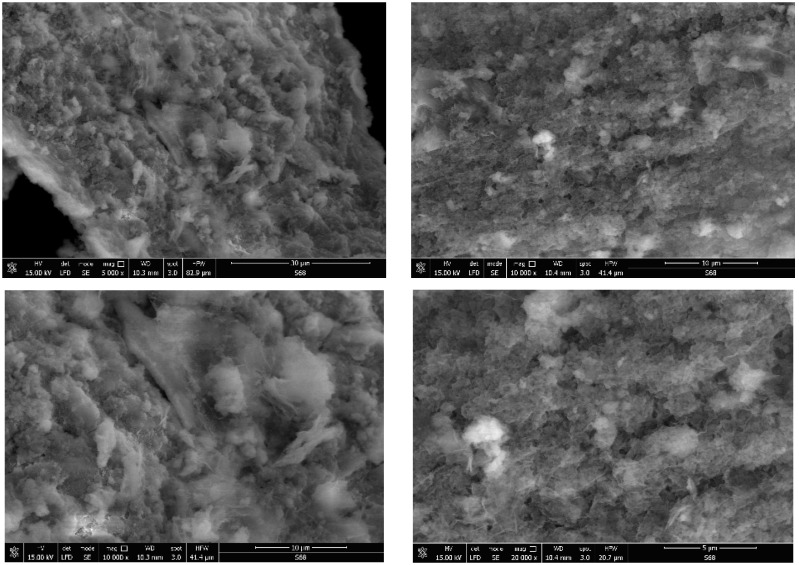
SEM images of the hybrid compound S2.

**Figure 4:**
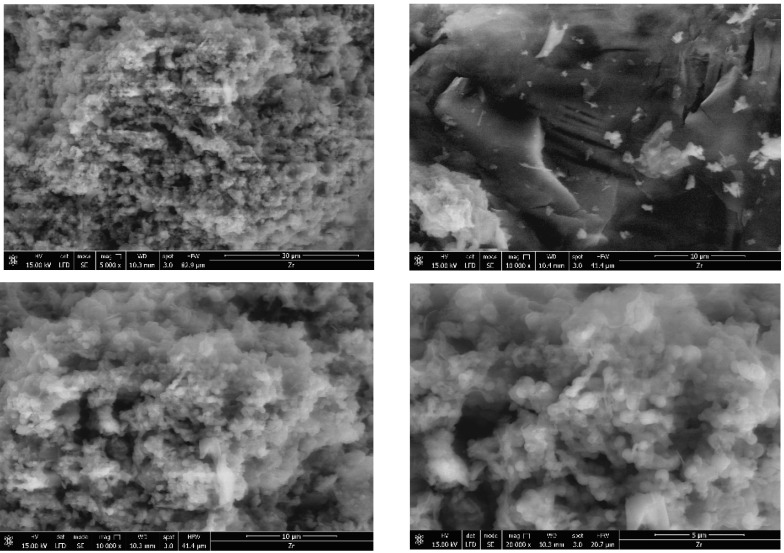
Results obtained by using SEM for hybrid S3.

**Figure 5:**
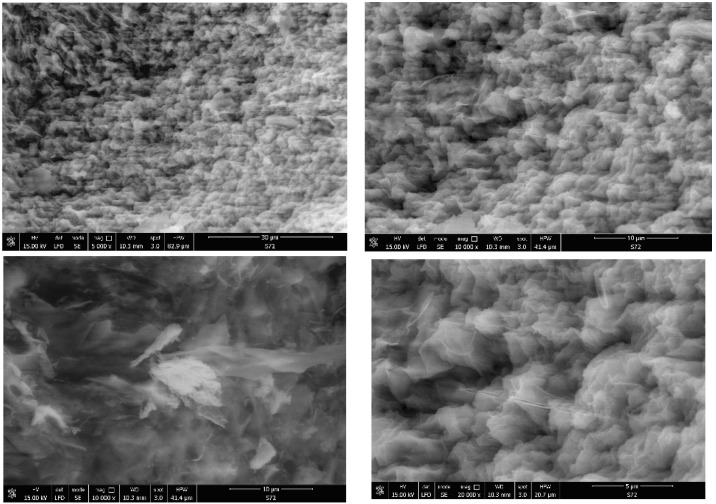
Hybrid S4 morphology observed with SEM.

**Figure 6:**
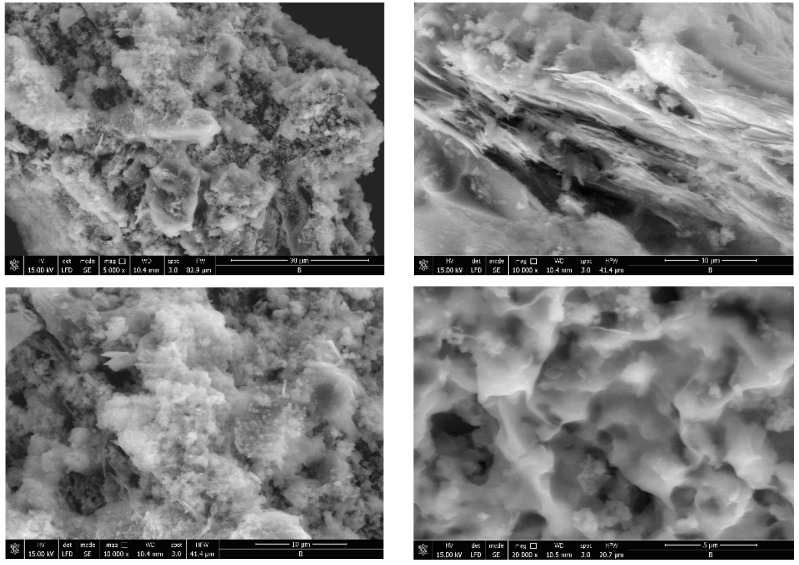
SEM results of hybrid S5 surface conformation.

The porosity of the obtained materials and the pore shape and number on their surface is strongly influenced by the phosphorus source and by the ratio of the used reagents. Most of the SEM images generally showed a similar morphology for the obtained hybrids, with several compact structures (Figure 2, Figure 3, Figure 4, Figure 5 and Figure 6).

Starting from the same reagents and precursors and using the same conditions, except the molar ratio and the presence (S4 and S5) or absence (S1–S3) of boron, we expected to obtain a relatively similar morphology from the SEM images at this resolution. But a difference was still observed for hybrids S4 and S5 (Figure 5 and Figure 6) containing boron, in addition to phosphorus and zirconium, in terms of surface morphology, in comparison with hybrids S1–S3 (Figure 2, Figure 3 and Figure 4). The SEM images of hybrids S4 and S5, especially at the maximum resolution possible in these determinations, showed a higher surface porosity. This is the effect of boron. It is practically due to the B-O-Zr bridges that occurred. Table 1 shows the bridges formed and expected, and the bonds which could not be obtained in the mild conditions used are shown for each of the synthesized hybrids.

As can be easily observed from Table 2, Zr and Zr, P and Zr, or B and Zr, are always connected via an oxygen atom in the structure of the obtained hybrids, and moreover, it is not possible to obtain direct bonds between these elements in the mild conditions used. It is also obvious from Table 2 that it was not possible to obtain bridges such as P-O-P and P-O-B. As a consequence, only zirconium could be involved in bridges with phosphorus and boron, via an oxygen atom, as already explained. For that reason, when boron is also present (S4 and S5), an excess of zirconium will be necessary, due to its possible presence in the bridges mentioned above.

On the other hand, if we compare the SEM images of hybrids S1–S3 which did not contain boron, it can be observed that the materials of S1 and S2 are rather similar, but hybrid S3 showed a somewhat different surface morphology, due to the zirconium excess and therefore due to the presence of more Zr-O-Zr bridges.

The structure of such hybrids is very complex and could be assimilated to a randomly coiled polymer, with a different number and different order of the X-O-P bridges (where X could be P, Zr and B). For that reason, such hybrids are also called inorganic polymers (or inorganic co-polymers). Such organic–inorganic networks are highly suited to forming supramolecular structures and could be used for several applications in different fields, such as catalysis, energy storage, medicine or supramolecular chemistry. The metal used has a significant influence on the properties of the synthesized materials, in addition to the P/M ratio. When using tetravalent metal phosphonates (as is the case for zirconium), a phosphorus excess is needed [11]. Then, M-O-P and M-O-M bridges are obtained and the presence of residual M-O-H, P-OH and P=O bonds can be observed. On the other hand, boron is not a metal. It is at the border between metals and non-metals (practically, it is the only non-metal from its group, which contains aluminium, for instance). Boron is very often called metalloid, but it still forms B-O-Zr bridges in the structure of hybrids S4 and S5. Therefore, this B-O-Zr bridge is obviously not a M-O-M bond, but it is obtained according to the same mechanism. On the other hand, the presence of boron could not lead to the obtaining of P-O-B bridges (as was observed in the case of zirconium, i.e., Zr-O-P). Boron is just before carbon in the periodic table of the elements. As expected, boron is similar to carbon in its ability to form stable covalent bonds in molecular structures and networks (but, of course, their number is different). It forms regular boron icosahedra. Boron is also a very hard material, and therefore, it is expected to bring even more mechanical resistance to the obtained hybrids S4 and S5, in addition to the presence of zirconium.

The organic–inorganic hybrid materials were already involved in many applications of great interest, such as ion-exchange and catalysis [11,29,40,41,42,43,44,45,46], photocatalysis [15,30,34,35,36,37,38,41], medicine, or for electrochemical devices and membranes with specific transport properties in separation and sensor technologies, or as a stationary phase in chromatography [40,41,42,43,44,45,46,47,48,49]. The applications of organic–inorganic hybrid materials containing phosphorus are in general determined by their high specific surface area, from the presence of residual acidic P-OH groups and/or of the flexible organic functional groups, and from the properties of the rigid inorganic part (strongly influenced by the used metal) [41,42,43,44,45].

The synthesized materials are also very stable from a thermal point of view and insoluble in organic solvents and in water. In addition to these properties, which make the material very stable from a chemical and physical point of view, the synthesized organic–inorganic hybrids (S1–S5) are also mechanically resistant due to the presence of zirconium. Zirconium has started to replace titanium in the last decades in hard-tissue medical prostheses due to its mechanical properties. It is found in natural sources that contain a small amount (1–2%) of hafnium, and together with titanium, these three elements appear in the same group of the periodic table (the fourth group). Therefore, it is expected to have similar properties (including mechanical resistance). Although titanium was already applied and used, including in such organic–inorganic hybrids, the interest in using zirconium increased in the last decade and is currently still growing.

**Figure 7:**
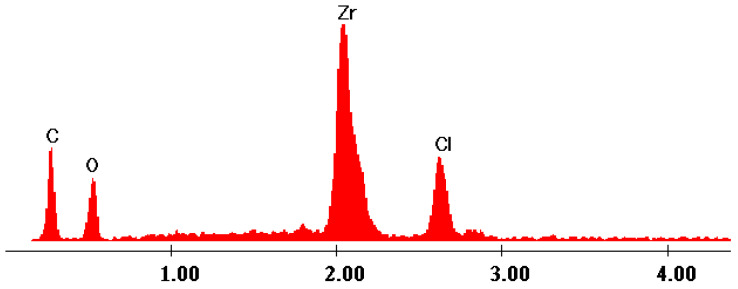
EDX data of hybrid S1.

**Figure 8:**
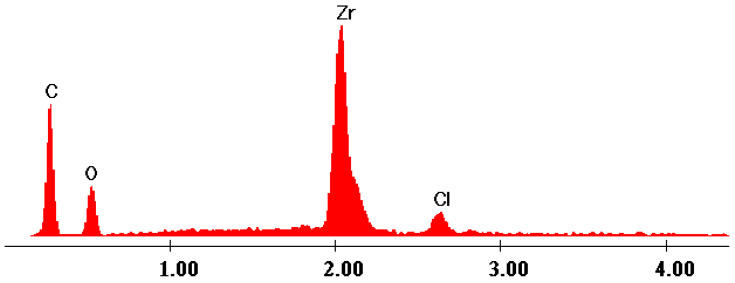
EDX data of hybrid S2.

**Figure 9:**
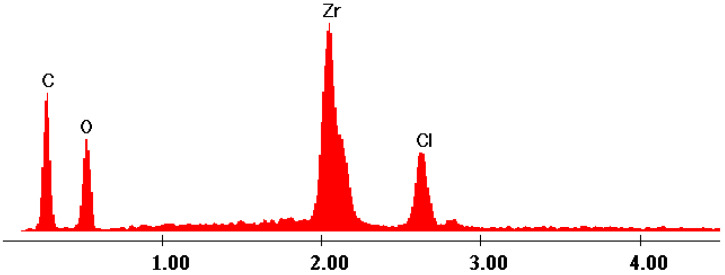
EDX data of hybrid S3.

**Figure 10:**
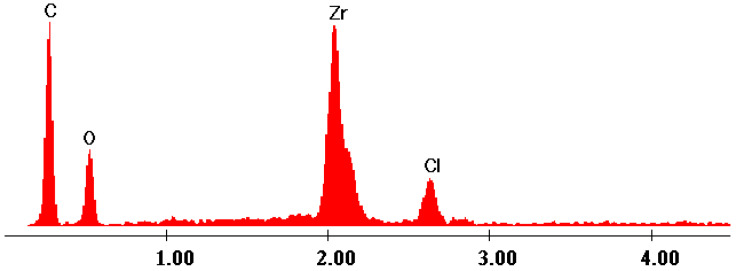
EDX data of hybrid S4.

**Figure 11:**
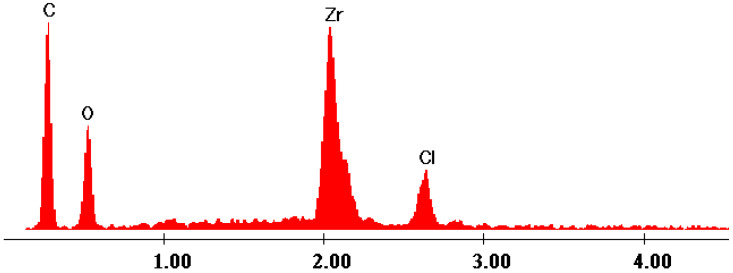
EDX data of hybrid S5.

The EDX data (Figure 7, Figure 8, Figure 9, Figure 10 and Figure 11) confirmed the presence of phosphorus and zirconium on the structure of the obtained hybrids. EDX analysis showed the presence of C, O, P and Zr for the performed sol–gel syntheses S1–S5.

The EDX data proved and confirmed that the chemical reactions performed by sol–gel method took place and the hybrid products were obtained as organic–inorganic networks. Table 2 presents the percentages of the content of the elements on the synthesized hybrids, from EDX data, according to Figure 7, Figure 8, Figure 9, Figure 10 and Figure 11.

The elemental composition was changed function of the molar ratio, as it is obvious from Table 3, and this is also confirmed from both SEM and EDX analyses. Moreover, a clear indication that the hybrid structures were formed is the fact that the product is not soluble and, during the synthesis, it started to form a sediment (and subsequently form a suspension). Moreover, all the used precursors are water soluble. Therefore, the initial mixture is a homogeneous solution, and the resulting product is a heterogeneous suspension containing the insoluble product, water and the dissolved (but not reacted) precursors and reagents (if it is the case, usually when an excess is used).

## 3. Conclusions

In the present work, novel hybrids containing zirconium and phosphorus compounds were synthesized by using the sol–gel method, starting from zirconyl chloride hexa-hydrate (ZrOCl_2_·6H_2_O) and phenyl phosphinic acid. All the syntheses were performed at room temperature by using water as solvent. The sol–gel syntheses were completed in 6 h. Afterwards, the synthesized hybrids were washed, subsequently filtered and finally dried in an oven at 80 °C for 8 h. The used reagents and precursors are water soluble, but the hybrid compounds are not. The obtained products are very stable from a thermal point of view and are not soluble. The sol–gel method is a green alternative for obtaining organic–inorganic hybrids containing phosphorus, zirconium and boron.

The chemical reaction takes place between phosphorous and zirconium or two zirconium atoms, always connected by an oxygen atom (P-O-Zr or Zr-O-Zr). The phenyl groups from the structure of phenyl phosphinic acid are very stable, very hydrophobic and do not react in the mild conditions used. These phenyl groups are found on the structures of the synthesized hybrids as well, connected in the phosphorus atom, as in the structure of phenyl phosphinic acid.

For hybrids S1–S3, EDX data confirmed the presence of phosphorus and zirconium on their structures. In addition, for hybrids S4 and S5, the presence of boron was proved. Moreover, SEM images showed similar morphology for all the hybrids synthesized in the present work, but some differences were observed when boron was used. When Zr was excessive, it was expected to have more Zr-O-Zr bridges than P-O-Zr bonds. The obtained hybrids have complex 3D structures and form organic–inorganic networks containing Zr-O-Zr, Zr-O-P and Zr-O-B bridges. It is very interesting that boron could be involved only in Zr-O-B bridges, while zirconium could be involved in all Zr-O-Zr, P-O-Zr and B-O-Zr bridges. Such organic–inorganic networks are expected to form supramolecular structures and to have many potential applications in different fields of great current interest such as catalysis, medicine (especially for hard-tissue reconstruction), agriculture, energy storage, fuel cells, sensors, electrochemical devices and supramolecular chemistry. In addition, all the hybrids synthesized here showed high thermal stability, which makes them very attractive materials for different applications and processes that take place at high temperatures.

## 4. Materials and Methods

Zirconyl chloride hexa-hydrate (ZrOCl_2_·6H_2_O), phenyl phosphinic acid and triethyl borate B(OEt)_3_ were purchased from Fluka.

The reagents zirconyl chloride hexa-hydrate (ZrOCl_2_·6H_2_O), triethyl borate and phenyl phosphinic acid were used at different molar ratios (Table 3).

The sol–gel method was used for obtaining the hybrid materials containing zirconium, boron and phosphorus compounds. All the syntheses were performed at room temperature by using water as solvent. After the syntheses were carried out, the obtained hybrids were filtered, washed and dried in an oven for 6 h at 80 °C. All the hybrid solid materials were further analyzed and characterized by using SEM and EDX methods. SEM and EDX measurements and determinations (including the images of the material surfaces and the element content) were performed by using a Jeol JSM 6400 Scanning Microscope coupled with an X-ray microanalyzer EXL II System Link Analytical, with a detector of 133 eV.

## Figures and Tables

**Table 1:** The bridges from the structures of the synthesized materials.

Bridge	S1	S2	S3	S4	S5
P-O-Zr	Expected	Expected	Expected	Expected	Expected
Zr-O-Zr	Expected	Expected	Expected	Expected	Expected
P-O-P	Not possible	Not possible	Not possible	Not possible	Not possible
Zr-O-B	-	-	-	Expected	Expected
P-O-B	-	-	-	Not possible	Not possible
P-P	Not possible	Not possible	Not possible	Not possible	Not possible
Zr-Zr	Not possible	Not possible	Not possible	Not possible	Not possible
B-B	Not possible	Not possible	Not possible	Not possible	Not possible
P-Zr	Not possible	Not possible	Not possible	Not possible	Not possible
P-B	Not possible	Not possible	Not possible	Not possible	Not possible
Zr-B	Not possible	Not possible	Not possible	Not possible	Not possible

**Table 2:** Carbon and zirconium content (Wt %) from EDX data for the synthesized materials.

Organic–Inorganic Hybrid	C%	Zr%
S1	52.07	17.26
S2	60.55	15.25
S3	53.74	12.9
S4	62.25	11
S5	58.59	11.72

**Table 3:** The used reagents for the sol–gel syntheses S1–S5.

Organic–Inorganic Hybrid	Phenyl Phosphinic Acid:ZrOCl_2_:B(OEt)_3_
S1	1:1:0
S2	2:1:0
S3	1:2:0
S4	1:1:1
S5	1:2:1

## Data Availability

The data presented in this study are available from the corresponding author upon request.
